# Editorial: Metabolic modulation of cellular function

**DOI:** 10.3389/fcell.2024.1395922

**Published:** 2024-03-18

**Authors:** Or Kakhlon, Ann Saada, Pablo V. Escriba

**Affiliations:** ^1^ Department of Neurology, The Anges Ginges Center for Human Neurogenetics, Hadassah-Hebrew University Medical Center, Jerusalem, Israel; ^2^ Faculty of Medicine, Hebrew University of Jerusalem, Jerusalem, Israel; ^3^ Department of Genetic and Metabolic Diseases, Hadassah-Hebrew University Medical Center, Jerusalem, Israel; ^4^ Laminar Pharmaceuticals, Palma de Mallorca, Spain; ^5^ Department of Biology, University of the Balearic Islands, Palma de Mallorca, Spain

**Keywords:** metabolism, metabolic pathways, drug discovery, cell fate, metabolomics

In this Research Topic, metabolic changes are described as sensors or mediators of biological processes in cells and organisms. These functions are performed by metabolites as substrates, products, and allosteric regulators of cellular enzymes and as substrates of post-translational and epigenetic modifications, enabling them to mediate cell signaling. This Research Topic showcases metabolites as a pool of molecules (metabolome) which regulate cell function, fate and structure. This definition implies that not all the constituents of pathways or mechanisms influenced by the metabolome are known *a priori*, i.e., these pathways/mechanisms are investigated by an *untargeted* approach. *Untargeted* studies can thus *generate* new hypotheses on how metabolites sense or mediate the phenotype(s) investigated, or even discover novel pathways converging to and diverging from known pathways. Alternatively, in *targeted* analyses, well-established metabolites and metabolic pathways are presumably implicated in new phenotypes. Only these metabolites are quantified to obtain steady-state levels and metabolic fluxes by mass spectrometry and isotope tracing, respectively. Targeted studies *test* hypotheses limited to specific research questions. Targeted and untargeted analyses may also be combined to unveil novel connections and networks involved in the phenotypes studied. Lastly, metabolite levels and fluxes are also diagnostic and efficacy biomarkers. Metabolic investigations involving hypotheses testing and biomarkers generation illustrate how metabolic changes can modulate cell function as represented in this Research Topic.

Three papers illustrate how metabolic reprogramming can directly determine cell fate with ensuing consequences on health and disease. Zhao et al. show that during acute kidney injury (AKI) many anabolic and catabolic pathways are modified. Beyond the reprogramming of well-known pathways such as fatty acid oxidation and glycolysis, AKI also implicates triglyceride overaccumulation, activation of polyol metabolism and amino acid and redox modifications. These broad metabolic alterations activate programmed cell death pathways: apoptosis, ferroptosis, necroptosis, pyroptosis and autophagic block. This review thus represents a hypothesis-generating study in which the involvement of broad-scale metabolic pathways serves as a fertile ground for the discovery of novel metabolic-based therapeutic interventions. Another interesting review linking metabolic reprogramming with programmed cell death is Yang et al. This review discusses Paneth cell (Lueschow and McElroy, 2020), epithelial cells in the intestinal crypt, which maintain intestinal health. Paneth cells are multi-functional cells regulating microbiota composition (by secreting the selective bactericidal α-defensin), and intestinal epithelial and stem cells death and growth. This review is unique to this Research Topic, inasmuch as it does not describe specific metabolic pathways, but rather modulation of intestinal microbiota, which, on its own, is a key systemic metabolic modulator. Commensal microbiota determine intestinal metabolic homeostasis by both expressing metabolic enzymes not encoded by the host and cross-feeding between bacterial stains which produces nutrients for host consumption [*e.g.*, acetate produced by *Bifidobacterium* is a substrate for butyrate produced by *F. prausnitzii* (Lee et al., 2018)]. The third paper focusing on cell fate modulation by metabolic reprogramming is Sukjoi et al. The authors have previously shown that holocarboxylase synthetase (HLCS) is overexpressed and pro-oncogenic in the triple-negative breast cancer (TNBC) cell line MDA-MB-231. Here they test the hypothesis that HLCS knockdown is anti-carcinogenic and investigate which biochemical and metabolic changes HLCS knockdown causes. They show that HLCS knockdown is mainly associated with inhibition of proteins facilitating invasiveness and metastasis, such as SerpinB2 (Harris et al., 2017) and collagenase (Fields, 2013). The authors also suggest that HLCS knockdown inhibits malignant cell growth by suppressing the urea cycle: HLCS-mediated increase in argininosuccinate synthase can increase aspartate and ensuing nucleotide synthesis and constitutive proliferation.

Two papers describe diseases directly caused by metabolic dysfunction, possibly ameliorated by endogenous metabolic factors. Bourebaba et al. show that sex hormone binding globulin (SHBG) can lower saturated and pro-inflammatory fatty acids causing metabolic syndrome. Furthermore, SHBG induced lipolysis, repressed lipogenesis, increased insulin sensitivity by boosting insulin signaling receptors and attenuated pro-inflammatory factors demonstrating a comprehensive effect on metabolic syndrome. Shilian et al. discuss the role of the elongator complex, deficient in familial dysautonomia (FD), on acetyl-CoA synthesis/target acetylation through stabilization of the key metabolic enzyme ATP-citrate lyase (ACLY) which acetylates the cytoskeleton enabling neuritogenesis. Elongator deficiency leads to proteasomal degradation of microtubule associated protein Tau, reversible by deacetylase inhibitors. Moreover, Tau and neuritogenesis deficiencies can be corrected by ACLY overexpression demonstrating a novel potential metabolic therapy for FD through acetyl-CoA restoration.

Another set of articles uses knowledge on metabolic reprogramming as a platform for drug discovery. Arévalo et al.’s review provides an updated account of the long-known anti-leukemic efficacy of botanical drugs. They show that chemoresistant metabolic reprogramming in leukemia cells (glycolytic activation and OxPhos suppression) is modified by botanical compounds, *e.g.*, inhibition of glycolytic enzymes by alkaloids and enhancement of fatty acid oxidation by avocado-extracted lipid B. These metabolic modifications predispose originally chemoresistant leukemia cells to cytotoxic chemotherapy making botanical drugs preferred co-adjuvants. The approach presented in this review is illustrated in Granit et al.’s research article. The authors tackle cisplatin chemoresistance in TNBC purportedly caused by metabolic and ensuing epigenetic reprogramming. They show that the histone deacetylase inhibitor valproic acid (VPA) can mitigate cisplatin chemoresistance by inhibiting fatty acid oxidation and OxPhos through decreasing both acylcarnitines and carnitine as an FAO precursor. In a follow-up work Mizrahi et al., the authors show that VPA actually increases cisplatin chemoresistance by up-modulation of glycolysis and the anti-oxidant enzyme aldehyde dehydrogenase (ALDH) and that only combining VPA with the pro-oxidant ALDH inhibitor disulfiram can overcome these pro-resistance effects of VPA both in TNBC cells and in more patient-relevant TNBC organoids. The work by Gharaba et al. uses aberrant cell-morphological phenotype as a hypothesis-driven strategy for potential drug discovery. Newly discovered deficient nuclear actin cap in Huntington Disease (HD) fibroblasts is associated with aberrant cell motility as acceptedly manifested in Hutchinson-Gilford progeria syndrome, used as an actin cap lacking reference. The authors propose that the quantifiable correction of aberrant actin cap and motility might serve to assess potential HD drug efficiency and demonstrate this by correction of these phenotypes using the mitochondrial antioxidant Mito Q.

Other papers provide metabolic underpinnings for key organismal processes, embryonal development and aging. Sakai et al. show that hypoxia-triggered and glycolysis-enabled proliferation is necessary for neural tube closure of neuroepithelial cells, which is not influenced by OxPhos modulation. Fang et al. describe systemic metabolic biomarkers of aging showing their association with attrition over time of mitochondrial TCA cycle-dependent pathways and parallel increase in glycolytic flux. Many of these biomarkers are accumulated metabolites which do not degrade. As opposed to the systemic level, at the organ level, inter-organ metabolomic variability is caused by differences in aging onset, *e.g.,* glycolysis induced at different ages in different muscle types.

Lastly, Esterhuizen et al.’s perspective reviews the impact of environmental stressors on metabolic diseases concluding that this relationship is bidirectional: environmental stressors suppressing human physiology, and physiological stress, especially perturbed hormonal balance and immunity, predisposing to environmental diseases, especially due to compromised detoxification capacity.

In summary, this Research Topic comprises a diverse set of papers on the modulation of cell function via metabolic reprogramming. Most papers test hypotheses and tackle the involvement of metabolic reprogramming in cell fate determination, endogenous pathogenic mechanisms, drug and therapy discovery and key biological processes. All papers showcase that a deep understanding of the metabolic machinery is key to the deciphering of biological processes in health and disease.
