# Corrigendum: Editorial: Metabolic modulation of cellular function

**DOI:** 10.3389/fcell.2024.1403128

**Published:** 2024-04-11

**Authors:** Or Kakhlon, Ann Saada, Pablo V. Escriba

**Affiliations:** ^1^ Department of Neurology, The Anges Ginges Center for Human Neurogenetics, Hadassah-Hebrew University Medical Center, Jerusalem, Israel; ^2^ Faculty of Medicine, Hebrew University of Jerusalem, Jerusalem, Israel; ^3^ Department of Genetic and Metabolic Diseases, Hadassah-Hebrew University Medical Center, Jerusalem, Israel; ^4^ Laminar Pharmaceuticals, Palma de Mallorca, Spain; ^5^ Department of Biology, University of the Balearic Islands, Palma de Mallorca, Spain

**Keywords:** metabolism, metabolic pathways, drug discovery, cell fate, metabolomics

In the published article, there was an error in the **Affiliation** list. Instead of “^1^Hadassah Medical Center, Jerusalem, Israel, ^2^University of the Balearic Islands”, it should be:

“^1^Department of Neurology, The Anges Ginges Center for Human Neurogenetics, Hadassah-Hebrew University Medical Center, Jerusalem, Israel

^2^Faculty of Medicine, Hebrew University of Jerusalem, Jerusalem, Israel

^3^Department of Genetic and Metabolic Diseases, Hadassah-Hebrew University Medical Center, Jerusalem, Israel

^4^Laminar Pharmaceuticals, Palma de Mallorca, Spain

^5^Department of Biology, University of the Balearic Islands, Palma de Mallorca, Spain.”

In the published article there was an error in the **Conflict of interest** section. It was missing the mention of Laminar Pharmaceuticals. This should have been written as:

